# Supplementary material for: Emotion dynamic patterns between intimate relationship partners predict their separation two years later: A machine learning approach
Source: PLoS One. 2023 Jul 6;18(7):e0288048. doi: 10.1371/journal.pone.0288048 (PMC10325102; doi:10.1371/journal.pone.0288048)

Supplementary Material

A) Positive interactions versus conflict interactions

Random Forest MMC

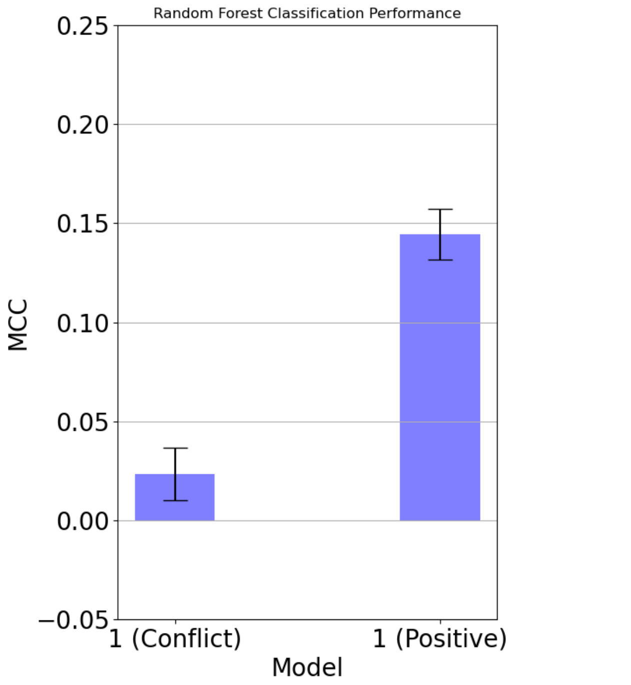

Random Forest MMC & demographics

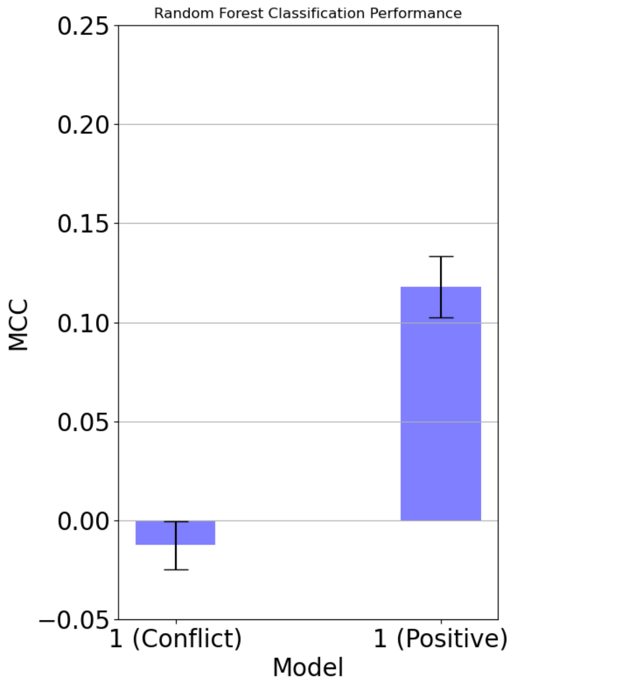

Random Forest AUC

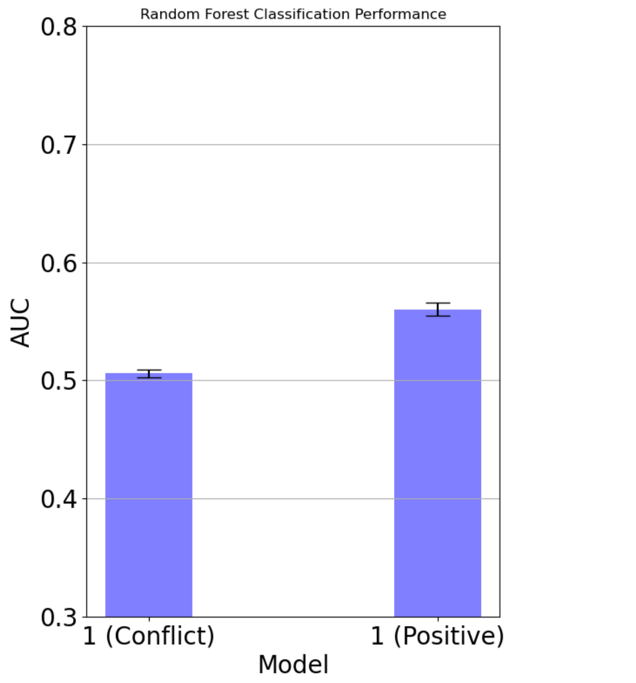

Random Forest AUC & demographics

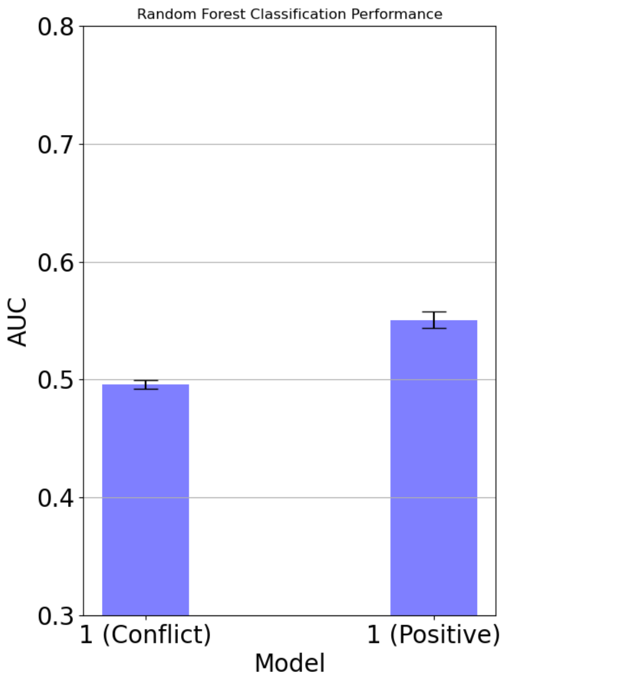

B) Figures for Positive interactions

Logistic Regression

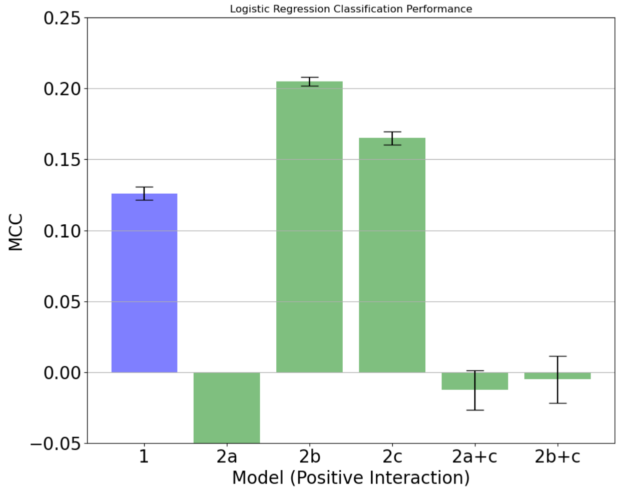

Logistic Regression & demographics

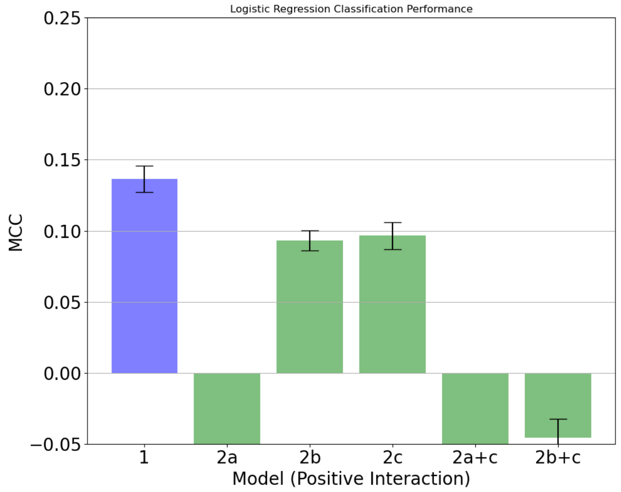

Random Forest

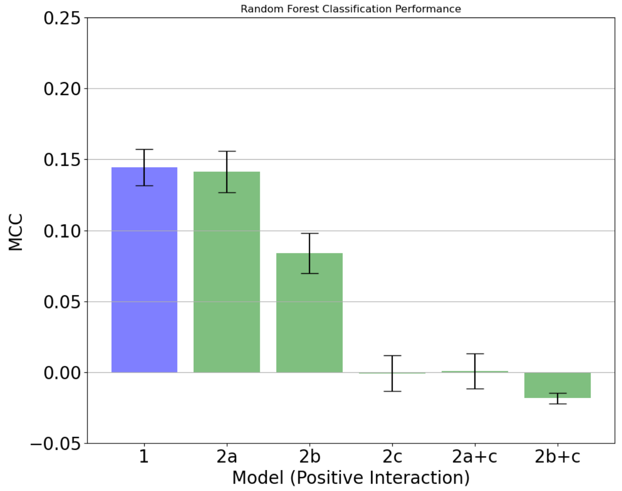

Random Forest & demographics

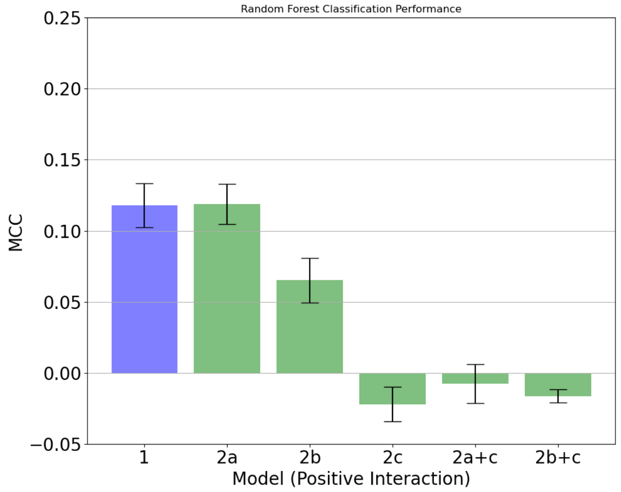

Random Forest & AUC

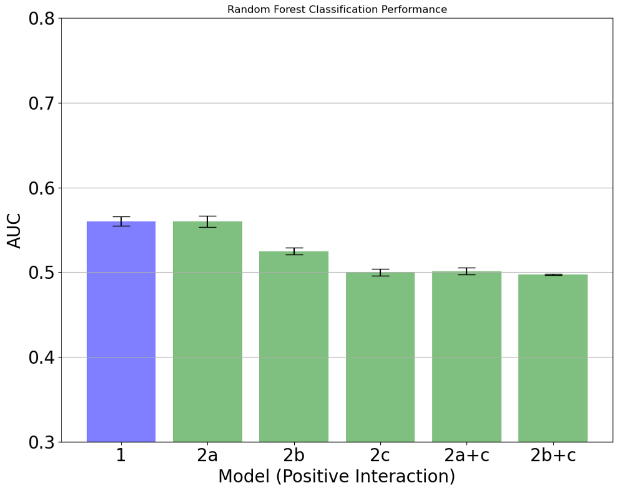

Random Forest & AUC & demographics

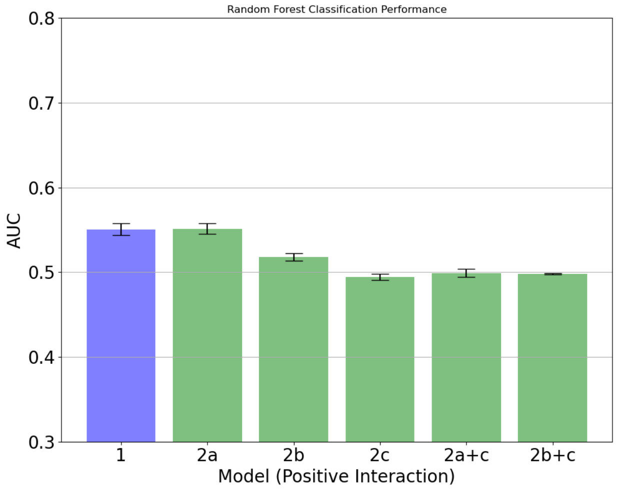

C) Figures for Conflict interactions

Logistic Regression

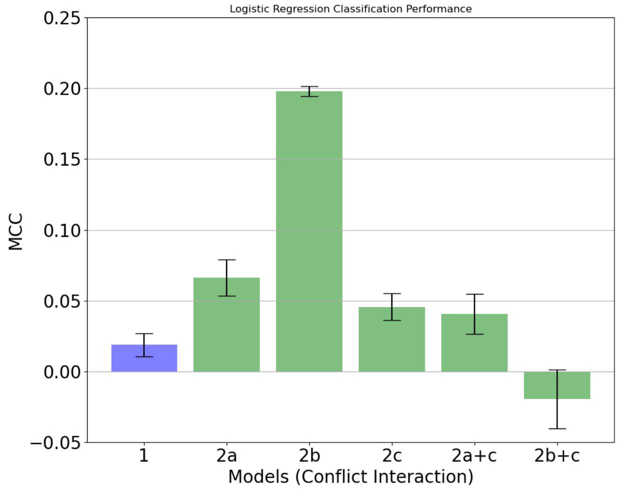

Logistic Regression & demographics

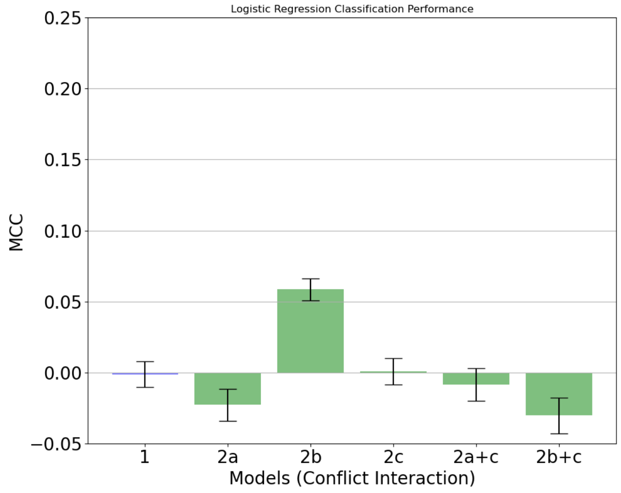

Random Forest

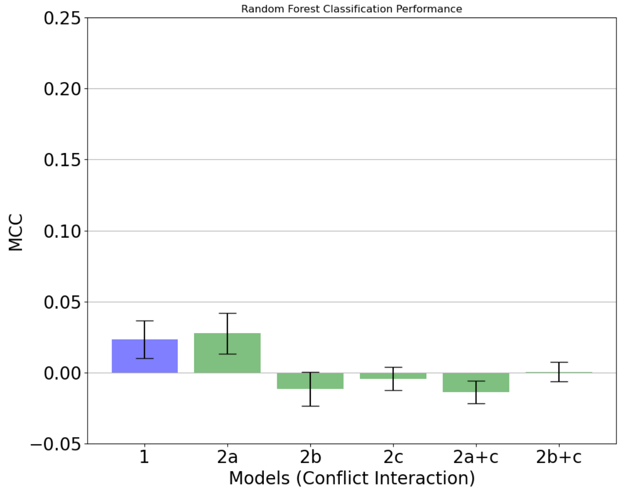

Random Forest & demographics

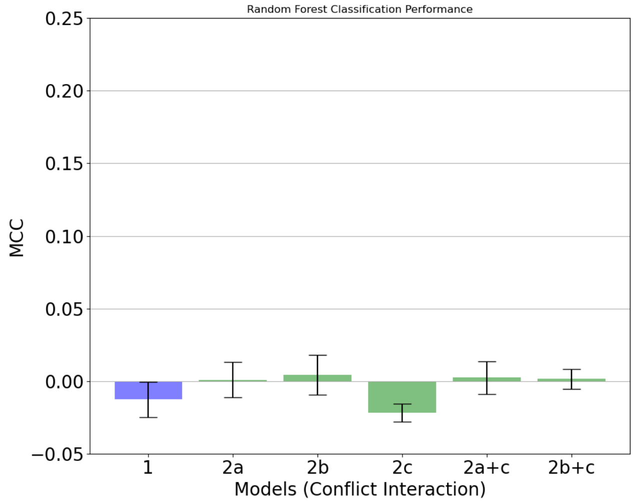

Random Forest & AUC

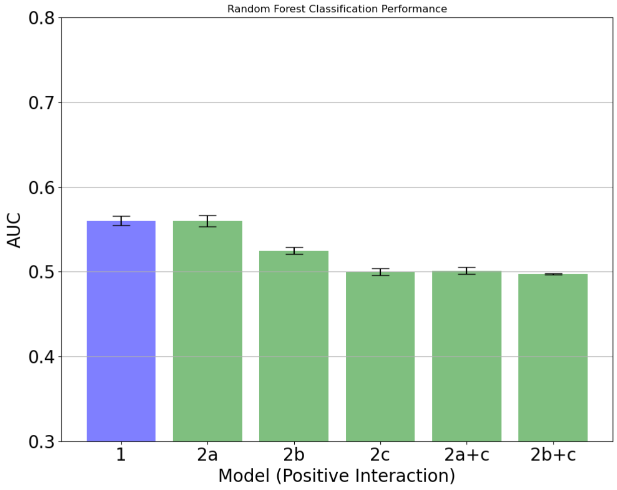

Random Forest & AUC & demographics

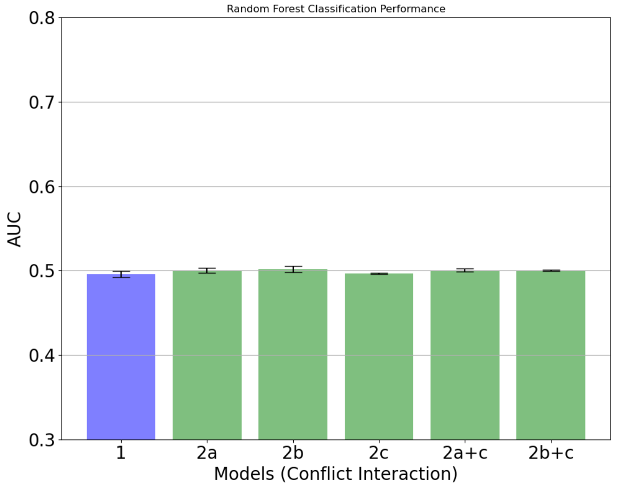

Supplement: S1 File — A) Posi4ve interac4ons versus conflict interac4ons. B) Figures for Posi4ve interac4ons. C) Figures for Conflict interac4ons. (PDF) [file pone.0288048.s001.pdf]
