# Supplementary material for: Emotion dynamic patterns between intimate relationship partners predict their separation two years later: A machine learning approach
Source: PLoS One. 2023 Jul 6;18(7):e0288048. doi: 10.1371/journal.pone.0288048 (PMC10325102; doi:10.1371/journal.pone.0288048)
Supplement: Attachment — Submitted filename: Letter_Reviewer.docx [file pone.0288048.s002.docx]

PONE-D-22-33994

Dear editor and dear reviewer,

Thank you very much for the suggestions. We tried to implement them as good as possible and hope that this has improved the manuscript.

Review Comments to the Author

Please use the space provided to explain your answers to the questions above. You may also include additional comments for the author, including concerns about dual publication, research ethics, or publication ethics. (Please upload your review as an attachment if it exceeds 20,000 characters)

Reviewer #1: The paper addresses a relevant question and is methodologically sound. I would recommend accepting the paper provided the following revisions are adequately addressed.

Major

The paper would benefit from showing additional benchmarks. For example, it is likely that demographic features and their interactions are predictive of relationship outcomes. Other information that was collected about participants could also be used to construct theory informed benchmarks. It would be interesting to see whether the emotion features would incrementally improve predictive performance when added to the same model (i.e. whether they capture non-redundant target variance). Being able to show that the emotion features capture non-redundant information would make the paper much stronger.

ANSWER

Thank you for this very interesting question. In fact, creating a comparison standard is an excellent way to test whether the variability in emotions plays a role or whether emotions could potentially be a proxy for other factors.

Therefore, we first calculated all models with the additional variables age (of each partner in the couple) and relationship living status (married, cohabiting, not cohabiting) to compare whether there was any difference, with the idea that if there were differences, we could proceed step by step to see which factors improve performance. More variables could not be included as this is a secondary data analysis and these are all the socioeconomic data we have access to.

However, the results show that the additional variables do not lead to a change in the results. In fact, we see some reduction in predictive performance. Whilst in our experience it is more common for the addition of information to improve predictive performance, we have sometimes found that, for datasets with small effects, the relationship is not always that of a monotonic improvement. In any case, the results indicate that the effect is explained by emotional experience and dynamics between partners, and that emotions contain non-redundant information. It could have been the case that emotions were only a proxy for other factors, but the additional analyses indicate that this is not the case.

Here are all the examples, which will be included into the supplementary material (on the right sight all are the figures including additionally the two demographic variables.

**Supplementary Material**

**A) Positive interactions versus conflict interactions**

| Random Forest MMC | Random Forest MMC & demographics |
| --- | --- |
|  |  |
| Random Forest AUC | Random Forest AUC & demographics |
|  |  |

**Overall, it appears that adding demographic variables has resulted in a slight decline in prediction accuracy, rather than an improvement.**

**B) Figures for Positive interactions**

| Logistic Regression | Logistic Regression & demographics |
| --- | --- |
|  |  |
| Random Forest | Random Forest & demographics |
|  |  |
| Random Forest & AUC | Random Forest & AUC & demographics |
|  |  |

**C) Figures for Conflict interactions**

| Logistic Regression | Logistic Regression & demographics |
| --- | --- |
|  |  |
| Random Forest | Random Forest & demographics |
|  |  |
| Random Forest & AUC | Random Forest & AUC & demographics |
|  |  |

It is clear that the demographic variables had no significant influence on the results. All these plots can be found in the supplemental materials.

This reinforces our confidence in the findings. It is actually quite remarkable that emotional information from just 10 minutes of interaction can predict separation two years later. We would not argue that the observed emotions cause separation, but it may serve as a proxy for how partners interact and experience each other, which is indicative of a potential future separation, and this relationship seems worthy of future replication and exploration.

We have added this clarification in the article.

Statistical Analysis

“As it is possible that emotional experience and dynamics between partners may capture redundant information, all models are compared with extended models that include demographic factors as predictors. This helps us determine whether emotions may capture redundant information.”

Results

“Finally, all models were then compared to models in which we included additional information regarding demographics. However, since there were no significant differences, these results have been included as supplemental material.”

COMMENT
The directionality of the association between emotion variability and relationship stability remains unclear and the authors suggest this as a direction for future research. I strongly recommend addressing this question in the current paper. This could easily be done by showing descriptive relationships between properties of the distribution of input features and the target variable. For example, the authors could compute point-biserial correlations between the person-level variance scores of emotion measures and the relationship outcome. For completeness this could also be done for the mean. Alternatively, the authors could show differences between the distributions of relationship outcome classes the same way it was done for CPSD (fig 4).

ANSWER

Thank you for this idea. We have already attempted to go beyond pure prediction and understand conceptually whether more or less variance predicts separation. This would be interesting to know because exploratively we could draw the conclusion whether too much emotional fluctuation or too little fluctuation might have something to do with later separation, even though this would only be on an exploratory level.

As suggested, we have examined the biserial correlations to see if there are any relationships between properties of the distribution of input features and the target variable. However, the results of the biserial correlations indicate no significant relationship between the variance of someone's time series and the breakup. All p-values are greater than 0.05. The result supports our previous statement that the reason for the relationship is unclear, and that further research is needed.

We have supplemented the manuscript with the table of correlations and provided a more detailed description.

«This indicates that the variability of emotions across the interaction did contain important information about whether or not couples will break up. As the variance information is at the individual level, rather than the couple level, it is non-trivial to identify whether, for instance, higher or lower variance is associated with breakup. For example, it may be that partner A has high variance, and partner B has low variance. Additional point-biserial correlations were computed between the individual emotion variability over a sequence and the breakup (Table 1). As the correlations were not significant, the direction of the relationship is not clear, indicating that variability alone is not sufficient in determining breakup. Further research is needed to specifically investigate the nature of the relationship between emotion variance and breakup.

Table 1. Point-biserial correlations examining whether the emotional variance of someone's time series correlates with whether they break up.

| Variance | Break-up | p-value |
| --- | --- | --- |
| Male, positive interaction | 0.10 | .310 |
| Female, positive interaction | -0.06 | .567 |
| Male, conflict interaction | 0.18 | .086 |
| Female, conflict interaction | 0.16 | .122 |

COMMENT

Minor

MCC is a relatively uncommon measure for classification performance. For completeness the authors should add the standard metrics (accuracy, precision, recall, and AUC) to the visualizations and consider reporting AUC as the main metric throughout the paper.

ANSWER

We agree with the reviewer that for individuals familiar with machine learning, AUC is the better approach (amongst others which the reviewer mentioned) to measure classification performance. However, our experience has shown that psychologists are often unfamiliar with AUC, as it is not part of the standard statistical training. Although MMC is also not a standard tool in the psychologist's repertoire, we have found that psychologists have a better understanding of MMC, as they are familiar with the concept of correlation. Therefore, to ensure the results are understandable for psychologists, we will maintain MMC in the manuscript. Nevertheless, we have also calculated the standard metric of AUC and present it in the supplemental materials.

COMMENT

The authors refer to their study as an experimental study on several occasions (e.g. page 21). This is imprecise as the study is clearly correlational and there was no randomly assigned manipulation. The authors should correct this.

ANSWER

In our opinion, it is an experiment because we have a manipulation - the interactions (positive interaction versus conflict interaction). Yes, there is no control group of other couples in this sense, but we can now compare the two interactions. This means that the control group for the positive interaction is the data from the conflict interaction and vice versa. This is the reason why we also compared then in this study. We believe that this is a very good control group and better than comparing it with other couples. Therefore, it is not actually an observation since we manipulated the interactions. We can change the terminology if the reviewer insists, but in our opinion, the term "observational data" is not correct in this case since we "forced" people into different interactions.

COMMENT

Formatting / style

ANSWER

We have reformatted the manuscript and made it compatible with PLOS ONE guidelines.

COMMENT

The following sentence is incomplete: “After classifying 10-second sequences as positive, negative, or neutral, they predicted partners’ next sequence the person’s prior sequence and found that unhappy couples experience greater reciprocity of negative affect (17,24) and high affect reciprocity 75 (12) than happy couples.”

ANSWER

We revised the sentence:

” After classifying 10-second sequences as positive, negative, or neutral, the researchers predicted their partners' next sequence based on their prior sequence. They found that unhappy couples exhibit greater reciprocity of negative affect [17], [24] and higher affect reciprocity compared to happy couples [12].”

COMMENT

The title on page 1 is different from the title on the cover page

ANSWER

Title: Emotion Dynamic Patterns Between Intimate Relationship Partners Predict Their Separation Two Years Later: A Machine Learning Approach
